# Differences in Exposure to Nicotine, Tobacco-Specific Nitrosamines, and Volatile Organic Compounds among Electronic Cigarette Users, Tobacco Smokers, and Dual Users from Three Countries

**DOI:** 10.3390/toxics8040088

**Published:** 2020-10-14

**Authors:** Danielle M. Smith, Lion Shahab, Benjamin C. Blount, Michal Gawron, Leon Kosminder, Andrzej Sobczak, Baoyun Xia, Connie S. Sosnoff, Maciej L. Goniewicz

**Affiliations:** 1 Department of Health Behavior, Roswell Park Comprehensive Cancer Center, Elm and Carlton Streets, Buffalo, NY 14263, USA; danielle.smith@roswellpark.org; 2 Department of Epidemiology and Public Health, University College London, 1-19 Torrington Place, London WC1E 6BT, UK; lion.shahab@ucl.ac.uk; 3 Tobacco and Volatiles Branch, Division of Laboratory Sciences, Centers for Disease Control and Prevention, Atlanta, GA 30341, USA; bkb3@cdc.gov (B.C.B.); Vvq2@cdc.gov (B.X.); css3@cdc.gov (C.S.S.); 4 Department of General and Inorganic Chemistry, Katowice Faculty of Pharmaceutical Sciences in Sosnowiec, Medical University of Silesia, Jagiellonska 4, 41-200 Sosnowiec, Poland; mch.gawron@gmail.com (M.G.); leon.kosmider@gmail.com (L.K.); asobczak@sum.edu.pl (A.S.)

**Keywords:** e-cigarettes, harm reduction, vaping, nicotine, toxicants, global health

## Abstract

Country-level differences in nicotine vaping products used and biomarkers of exposure among long-term e-cigarette users and dual users remain understudied. This cross-sectional study was conducted in 2014 in the United States (*n* = 166), United Kingdom (*n* = 129), and Poland (*n* = 161). We compared patterns of tobacco product use and nicotine and toxicant exposure among cigarette-only smokers (*n* = 127); e-cigarette-only users (*n* = 124); dual users of tobacco cigarettes and e-cigarettes (*n* = 95); and non-users (control group, *n* = 110) across three countries using mixed-effects linear regression. Compared with cigarette smokers, e-cigarette-only users had lower levels of toxicant biomarkers, but higher levels of nicotine biomarkers. Dual users had higher levels of toxicant biomarkers than e-cigarette-only users but similar levels to cigarette-only smokers. E-cigarette users in Poland, who overwhelmingly used refillable tank devices, exhibited greater levels of nicotine, and toxicant biomarkers relative to e-cigarette users in US/UK. Despite smoking fewer cigarettes, dual users from Poland exhibited similar levels of nicotine biomarkers compared with UK dual users, but higher than US dual users. Country-level differences in e-cigarette devices used and smoking behaviors (e.g., intensity) may contribute to differences in biomarker levels among users of the same products residing in different countries.

## 1. Introduction

Worldwide, electronic cigarettes (e-cigarettes) have become increasingly popular [1]. Proponents of e-cigarettes note that these devices may have public health benefits, since these products deliver nicotine to users without exposing them to high doses of potential toxicants such as tobacco-specific nitrosamines (TSNAs) and volatile organic compounds (VOCs) [2,3,4]. Although toxicant exposure is significantly reduced in exclusive e-cigarette users compared with cigarette smokers, e-cigarettes expose users to low, but non-negligible levels of several cardiovascular and respiratory toxicants, including formaldehyde, acetaldehyde, acrolein, benzaldehyde, metals, and particulates. Additional concerns surround users of both cigarettes and e-cigarettes (dual users), who appear to sustain or even increase their nicotine intake without any significant reduction in toxicant exposure compared with cigarette smokers [3].

Safety concerns have emerged regarding toxicant levels in e-cigarette emissions, in light of research showing the presence of contaminants in the products, and generation of thermal degradation byproducts of nicotine solvents during use [5]. High quantities of those thermal degradation byproducts—including formaldehyde, acetaldehyde, and acrolein—have been shown to be generated in high-powered devices. It also appears that there is an interaction between device characteristics, nicotine content in e-cigarette liquid, user puffing behaviors, and exposure to toxicants. For example, a recent study by Dawkins et al. has shown that use of a lower nicotine concentration e-cigarette liquid may be associated with compensatory behavior (e.g., higher number and duration of puffs) and increases in formaldehyde exposure [6]. Complex interactions between patterns of use for e-cigarettes and conventional cigarettes, and exposure to toxicants also exist among dual users. Our recent study has showed that cigarette smoking frequency appears to be the primary driver of toxicant exposure among dual users, with little-to-no effect of e-cigarette use frequency [7].

Most studies that examine nicotine exposure in exclusive e-cigarette users and dual users are limited to a single geographic location, failing to account for potential differences that may occur based on background environmental exposures, product availability, norms, and policies in different nations [3,4]. O’Connor et al. found that the type of e-cigarette device used by vapers from the US, England, Canada, and Australia differed by pattern of use and country. Exclusive, daily vapers were more likely to use refillable pen-shaped or box-shaped devices than disposable cig-a-like devices, when compared with other (non-daily/dual) users. Cartridge-based (closed) systems, typically marketed by tobacco companies, were more common among respondents from the US and England [8].

It is currently unclear whether e-cigarette users who live in countries that have imposed maximum allowable nicotine concentration in e-cigarette liquids (e.g., EU) are exposed to lower levels of nicotine as compared to users who live in countries where highly concentrated nicotine solutions for e-cigarettes are widely available (e.g., US). This demonstrates a need to evaluate exposure to nicotine and toxicants in long-term single or dual users of electronic and conventional cigarettes across multiple countries. This study investigated a broad range of biomarkers of exposure to nicotine and toxicants in long-term users of nicotine-containing products. The research addressed four main questions: (1) Is nicotine intake significantly different between long-term users of cigarettes, e-cigarettes, and both products from different countries? (2) Are long-term exclusive users of e-cigarettes in different countries exposed to similar levels of toxicants? (3) How does exposure to nicotine and toxicants among long-term exclusive e-cigarette users from different countries differ from exposure among exclusive smokers and non-users who live in the same country? and (4) How does exposure to nicotine and toxicants among long-term dual users of e-cigarettes and cigarettes from different countries compare to exposure among exclusive smokers who live in the same country?

## 2. Materials and Methods 

### 2.1. Procedures and Participants

Using data from a cross-sectional study published by Shahab et al. [4], we performed a secondary analysis of data comparing nicotine and toxicant exposure among 456 participants from four distinct groups: (1) tobacco-only smokers (*n* = 127); (2) e-cigarette-only users (*n* = 124); (3) dual users who smoked tobacco cigarettes and also used e-cigarettes (*n* = 95); and (4) non-smoking, non-e-cigarette users (*n* = 110). The study was conducted in 2014 at three sites located in the United States (*n* = 166), the United Kingdom (*n* = 129), and in Poland (*n* = 161). Table 1 displays the distribution of each tobacco use group according to country.

Participants were recruited from local metropolitan areas using advertisements and underwent a telephone screening to determine eligibility and classification into one of the study groups based on self-reported behavior within the six-month period preceding their visit. “Non-smoking, non-e-cigarette users” (non-users) reported no use of any nicotine-containing products within the past six months. “Cigarette smokers” reported daily cigarette smoking for at least the past six months and smoked at least 5 cigarettes per day (CPD). “E-cigarette-only users” reported daily e-cigarette use for at least the past 6 months and used at least 10 nicotine-containing cartridges per week or 2 bottles of nicotine solutions or at least 5 disposable products per week. “Dual-users” reported a minimum e-cigarette use of 5 nicotine-containing cartridges/one bottle of nicotine solution/2 disposable e-cigarettes per week and smoked at least 2 CPD for at least the past six months. Additional inclusion criteria included: must be 18 years of age or older, report no current or past year incidence of kidney disease, and no serious psychiatric conditions. Exclusion criteria included pregnancy or current breastfeeding; inability to communicate in the primary language at the study site; or current use of smokeless tobacco, pipes, or cigars. Study activities involved a one-time visit lasting approximately one hour; participants were asked to refrain from eating food, consuming alcohol, or using the lavatory in the hour preceding their visit. At the visit, all participants provided written consent, completed a brief questionnaire, underwent an exhaled breath carbon-monoxide (CO) test (Bedfont Micro Smokerlyzer), and provided a urine sample. Urine samples were collected using a sterilized, sealable cup and were subsequently transferred into cryovials by study staff. All urine samples were shipped on dry ice to the U.S. Centers for Disease Control and Prevention (CDC) for laboratory analyses. Upon completion, participants were compensated for their time. All methods and procedures were approved by the Institutional Review Boards at Roswell Park Cancer Institute in USA, University College London in UK, and Medical University of Silesia in Poland.

### 2.2. Questionnaire Measures 

Participants were asked questions about demographic characteristics (including age, sex, race, and education). Due to between-country differences in education, we grouped participant education into two distinct categories: low (equivalent to high school graduate) and high (equivalent to a college education). E-cigarette-only users, cigarette smokers, and dual users were asked about current patterns of e-cigarette and/or tobacco cigarette use (including the number of puffs taken per day on an e-cigarette, type of e-cigarette used, nicotine concentration used, and/or CPD). Due to differences in questionnaire design, we estimated the number of puffs taken per day on an e-cigarette for UK participants by taking the reported number of e-cigarette cartridges used per week multiplied by 200 (average puffs per cartridge), then divided this figure by 7 (days per week) [9,10]. At the time of the study, ‘pod mod’ style e-cigarettes (e.g., JUUL) were not available for purchase. 

### 2.3. Biomarker Measurements

All urine samples were analyzed by the CDC using mass spectrometric methods as previously described for 7 metabolites of nicotine [11], 18 metabolites of 14 VOCs [12], 5 metabolites of TSNAs, [13], and 2 minor tobacco alkaloids [11]. Analytic limits of detection (LOD) for each biomarker can be viewed in Supplementary Materials Table S1. Concentration of total nicotine equivalents (TNE-7) was calculated as the molar sum of total nicotine, cotinine, trans-3′-hydroxycotinine (including free form and their respective glucuronides); nicotine *n*-oxide and cotinine N-oxide, norcotinine, and nornicotine (nmol/mg creatinine).

### 2.4. Statistical Analysis

The sample size was determined using NNAL as the primary outcome measure to detect between-group differences according to tobacco use. We determined that a total of 180 participants (45 per group) would provide 80% power to detect between-group differences in NNAL levels with a moderate effect size of f = 0.25 [14]. Analyses examining country-level differences in biomarker concentrations across tobacco use groups were secondary, and should be considered exploratory. Sample characteristics were assessed for potential differences between study groups and between countries. Analysis of variance (ANOVA) was used to compare demographic characteristics for continuous variables, and Pearson χ^2^ tests were used to compare characteristics for categorical variables. For the main analyses, biomarker levels below the limit of detection were entered using a common substitution method (analyte-specific LOD/√2), [15] and were corrected for creatinine concentration. All biomarker data had skewed distributions and were transformed using the natural log. Adjusted geometric means were calculated to minimize the effect of skewness in the data on estimates and are reported for each assessed biomarker. Mixed-effects linear regression models were used to test between-group and between-country differences in urinary biomarkers using the log-transformed data. Models were adjusted for age, sex, and ethnicity (all fixed effects), while country was included as both a fixed and random effect. Pairwise comparisons were performed to assess country-level differences in biomarker levels across tobacco use groups (non-smokers, e-cigarette-only users, cigarette-only smokers, dual users) and within groups. Reported *p*-values were adjusted for multiple comparisons using Sidak’s method and were considered statistically significant at *p* < 0.05. In descriptive analyses, missing data were handled using listwise deletion; models were fit using maximum likelihood estimation. All analyses were conducted using Stata v.16.1.

## 3. Results

### 3.1. Demographics

Comparisons of the pooled sample demographics by tobacco use status showed significant differences by age and sex across non-users, e-cigarette users, dual users, and cigarette smokers (data not shown). Specifically, dual users were significantly younger than exclusive cigarette smokers (mean age: 37 ± 14 vs. 43 ± 14, *p* < 0.05), but were statistically similar in age compared to exclusive e-cigarette users and non-users (data not shown). The pooled sample was equally balanced in terms of demographics, socio-economic status and tobacco use groups across countries and the mean age (±SD) of participants was 40 ± 14 years (data not shown). Across countries, all participants differed by sex (*p* = 0.006) and race (*p* < 0.001, data not shown). US participants were significantly older than those in the UK or Poland (US = 45 ± 14, UK = 36 ± 12, PL = 38 ± 12 years (mean ± SD), F(2,453) = 18.775, *p* < 0.001, Table 1). UK e-cigarette-only users were significantly more likely to be male compared to Polish e-cigarette-only users, while all e-cigarette and dual users within Poland identified as being White, unlike counterparts in the US and UK (Table 1).

### 3.2. Patterns of E-Cigarette Use and Cigarette Smoking across Countries

Nearly three-quarters of all participants who reported using e-cigarettes (both exclusive and dual users) used refillable tank model e-cigarettes; 70% reported use of medium (10–15 mg) or high (16–24 mg) nicotine content products and averaged 90 ± 12 puffs per day on an e-cigarette. All cigarette smokers (exclusive and dual users) reported smoking an average 13 ± 9 CPD; dual users smoked significantly fewer CPD compared to exclusive smokers (mean CPD: 10 ± 8 vs. 15 ± 9, *p* < 0.001). E-cigarette-only users: UK e-cigarette-only users were less likely to report using refillable tank model e-cigarette devices than those in the US or Poland (Table 1). US e-cigarette-only users reported more puffs per day than those residing in Poland. Self-reported nicotine content did not differ by e-cigarette type or country. Dual users: UK dual users were less likely to report using refillable tank models than those in the US or Poland but were more likely to report using disposable and cartridge-based models. No differences in number of puffs on e-cigarette per day were detected in dual users across countries. Again, self-reported nicotine content did not differ by e-cigarette type or country. UK dual users reported higher CPD than dual users from the US or Poland. Cigarette smokers: In general, exclusive cigarette smokers smoked more CPD than dual users (t(216) = 4.939, *p* < 0.001). No statistically significant differences in cigarette consumption were observed in cigarette-only smokers according to country.

### 3.3. Nicotine Intake

**Figure 1:**
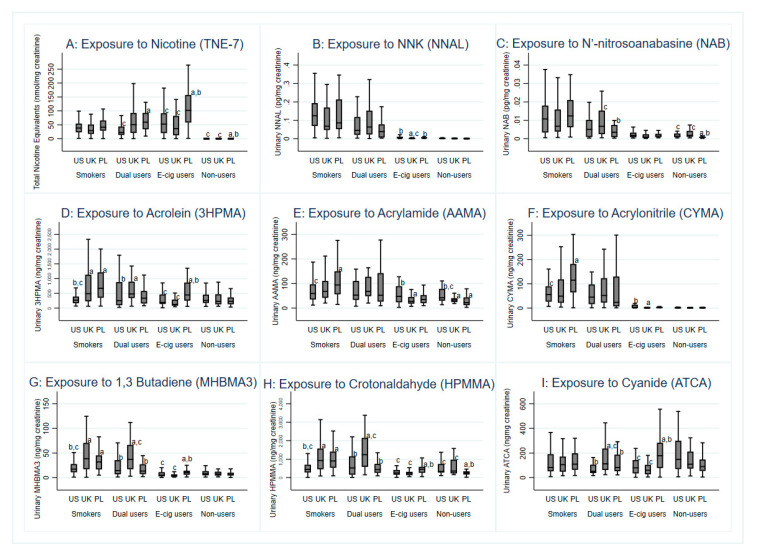
Biomarkers of exposure (**A**) nicotine, (**B**) tobacco-specific nitrosamine NNK, (**C**) N’-nitrosoanabasine, (**D**) acrolein, (**E**) acrylamide, (**F**) acrylonitrile, (**G**) 1,3-butadiene, (**H**) crotonaldehyde, and (**I**) cyanide; by tobacco use status and country of residence (*n* = 456). Superscript letters denote within-group differences by country of residence. a = significantly different from US; b = significantly different from UK; c = significantly different from Poland (PL) (according to sidak adjusted *p*-value < 0.05).

E-cigarette-only users: Adjusted models showed that e-cigarette-only users had significantly higher levels of several nicotine metabolites compared with dual users and cigarette-only smokers (all *p* < 0.05; see Supplementary Materials Table S2). We detected significantly higher levels of TNE-7, cotinine, and 3-hydroxycotinine in e-cigarette-only users compared to cigarette-only smokers. Figure 1A displays the distribution of TNE-7 according to tobacco use and country of residence. Polish e-cigarette-only users showed significantly higher levels of all biomarkers of nicotine intake compared to US and UK e-cigarette-only users (Table 2). No differences in nicotine exposure were observed between US and UK e-cigarette-only users. Dual users: Dual users showed comparable levels of nicotine metabolites as cigarette-only smokers and lower than e-cigarette users only (Figure 1A). Polish dual users also showed higher levels of several nicotine biomarkers compared to dual users from the US, but not from the UK (Table 3). Cigarette smokers: Cigarette smokers from all countries had similar levels of nicotine biomarkers, with the exception of Polish smokers, who had higher levels of unmetabolized nicotine and two minor nicotine metabolites (nicotine 1′-oxide and nornicotine) compared with US smokers (Table 4). 

### 3.4. Toxicant Exposure

Figure 1B–I displays the distribution of nine selected biomarkers of exposure, urinary NNAL and NAB (indicating exposure to tobacco specific nitrosamines), 3HPMA (acrolein), AAMA (acrylamide), CYMA (acrylonitrile), MHBMA3 (1,3-butadiene), HPMMA (ecrotonaldehyde), and ATCA (cyanide) according to tobacco use and country of residence. Statistical differences according to tobacco use can be viewed in Supplementary Materials Tables S2–S4. 

E-cigarette-only users: E-cigarette-only users had significantly higher levels of minor tobacco alkaloids (*p* < 0.001 Supplementary Materials Table S2); one tobacco specific nitrosamine (NNAL, *p* < 0.05, Supplementary Materials Table S3), and the biomarkers for acrylonitrile (CYMA, *p* < 0.001) and N,N-dimethylformamide (AMCC, *p* < 0.05, Supplementary Materials Table S4) compared with non-smoking controls (Figure 1B). E-cigarette-only users had significantly lower exposure to VOCs (biomarkers for acrolein, acrylamide, acrylonitrile, butadiene, crotonaldehyde, dimethylformamide, styrene, and xylene; all *p* < 0.05); and TSNAs (biomarkers of NAB, NAT and NNAL; all *p* < 0.05) than cigarette-only smokers (Figure 1 and Supplementary Materials Table S3).

US and Polish e-cigarette-only users exhibited significantly greater levels of urinary NNAL than UK e-cigarette-only users (*p* < 0.001; Table 2). UK e-cigarette-only users had significantly lower levels of urinary NNAL compared to those residing in the US and Poland. Polish e-cigarette-only users also showed higher levels of several VOC biomarkers, including acrolein, 1,3-butadiene, crotonaldehyde, cyanide, styrene, and ethylbenzene compared to US and UK e-cigarette-only users. US e-cigarette-only users also showed higher levels of biomarkers for acrylamide, acrylonitrile, N,N-dimethylformamide, and xylene than UK e-cigarette-only users (*p* = 0.05; Table 2). 

Dual users: We did not observe any statistically significant differences in minor tobacco alkaloids or most measured VOCs between cigarette-only smokers and dual users. As an exception, we noticed significantly lower levels of acrylonitrile (CYMA) among dual users compared with cigarette-only smokers (all *p* < 0.05; Figure 1 and Supplementary Materials Table S4). Three TSNAs (NNAL, NAT, NAB, all *p* < 0.05; Figure 1B and Supplementary Materials Table S3) were significantly lower among dual users compared with cigarette-only smokers. No differences in urinary NNAL levels were detected among dual users according to country; however, UK dual users had higher levels of NAB and NAT compared with Polish dual users (Table 3). UK dual users also showed higher levels of biomarkers for 1,3-butadiene, crotonaldehyde, and cyanide compared with US and Polish dual users. Additionally, UK dual users had higher levels of biomarkers for acrolein, styrene, and toluene than US dual users, and higher levels of biomarkers for acrylamide and xylene than Polish dual users. 

Cigarette-only smokers: Smokers across countries had similar levels of NNAL (Table 4). UK smokers showed higher levels of several VOC biomarkers than US smokers, including metabolites of acrolein, 1,3-butadiene, crotonaldehyde, and styrene. UK smokers also showed higher levels of metabolites of styrene compared with Polish smokers, but Polish smokers had higher biomarker levels of acrolein, acrylamide, acrylonitrile, 1,3-butadiene, crotonaldehyde, styrene, and ethylbenzene compared with US smokers. 

## 4. Discussion

This study provides novel information on exposure to nicotine, TSNAs, and selected VOCs in long-term exclusive or dual users of electronic and conventional cigarettes in three countries. Our findings indicate that exclusive e-cigarette users and dual users in Poland tended to exhibit greater concentrations of nicotine relative to counterparts in the US or UK Exclusive e-cigarette users in Poland also tended to exhibit greater exposure to a number of VOCs (including acrolein, cyanide, and 1,3-butadiene) relative to those in the US and UK The pattern of differences in biomarker concentrations between exclusive e-cigarette users, dual users, exclusive cigarette smokers, and non-users were relatively similar within each country. A novel finding is that e-cigarette users showed significantly higher levels of biomarkers of nicotine exposure than cigarette smokers. Previous studies suggest that exposure to nicotine from e-cigarettes is lower or equivalent to exposure from conventional cigarettes [2,16]. The increased exposure to nicotine observed in our study may potentially be explained by the observation that a significant proportion of e-cigarette users in our study used refillable tank-style devices. Also, many users reported using “other” types of vaping products, at least some of which may have used high-powered mod systems. This newer generation of vaping devices has been shown to deliver more nicotine to users than earlier generations of cig-a-like models [17]. 

To further explore this hypothesis, we conducted a post-hoc analysis of nicotine biomarkers among users of different types of e-cigarettes (Supplementary Materials Table S5). Despite the small sample of exclusive e-cigarette users who used replaceable cartridge models (cig-a-likes), we observed a trend suggesting that users of refillable tanks and ‘other’ devices had higher levels of nicotine metabolites in their urine. Additionally, users of ‘other’ devices showed significantly higher levels of nicotine metabolites compared to users of refillable tanks models. Those observations are consistent with findings that almost all e-cigarette users who lived in Poland used refillable tank systems, and the same group showed significantly higher levels of several nicotine metabolites compared to users from different countries. Future observational studies with larger sample sizes could verify whether potential differences in nicotine exposure are indeed driven by the popularity of e-cigarette devices with highly effective nicotine delivery, including the newest generation of salt-based pod systems.

Urinary levels of most toxicant biomarkers measured in this study did not differ significantly between e-cigarette-only users and non-users, except for a small number of VOCs and TSNAs. Our findings also showed that while being exposed to high nicotine levels, long-term exclusive e-cigarette users showed significantly lower levels of toxicant biomarkers compared with cigarette smokers or dual users. These results are consistent with a number of studies examining toxicant levels in e-cigarette users in contrast to conventional tobacco users [1,3,4,16,18,19,20]. In line with prior studies, our findings confirm that while not without some low level risk of exposure, exclusive e-cigarette use results in lower VOC and TSNA exposure compared with use of tobacco cigarettes and dual use.

Our novel observations related to differences in toxicant exposure among exclusive e-cigarette users from different countries may be a function of country-level differences in e-cigarette device availability, brand visibility, and consumer preferences. First, second, and third generation e-cigarette devices differ in the emissions of toxicants [1]. While our sample size was small, results suggest that cross-country comparisons of exposure to toxicants among e-cigarette users or dual users should control for device type. Differences in e-liquid manufacturing practices and quality control standards across countries could also potentially contribute to differences in toxicant exposure among e-cigarette users and remains an important area for future research. 

Results also suggest that while many biomarker levels were statistically similar between dual users and cigarette-only smokers, significantly lower levels for selective smoke exposure biomarkers (TSNAs and CYMA, a metabolite of acrylonitrile) were observed among dual users compared to smokers. Considering that dual users in our sample smoked fewer cigarettes overall compared with cigarette-only smokers, we suggest that this may be a function of differences in CPD among this group. This hypothesis appears to be consistent with observed differences in biomarkers levels in dual users from different countries: UK dual users, who smoked more CPD compared to Polish dual users, showed statistically higher levels of exposure biomarkers to several toxicants, including NNK, NAB, NAT, acrolein, acrylamide, benzene, 1,3-butadiene, crotonaldehyde, cyanide, styrene, and xylene. Therefore, it would appear that some cases of dual use may be associated with reduced exposure to some tobacco-related toxicants, depending on the relative frequency of e-cigarette use versus cigarette smoking, the number of cigarettes smoked, and type of e-cigarette device used [3]. However, our findings also clearly underscore the importance of complete smoking abstinence in achieving maximum exposure reduction to such toxicants. Taken together, compared with dual use of both e-cigarettes and cigarettes, this would indicate that complete substitution of e-cigarettes for cigarettes would yield an exposure reduction benefit for individual smokers. Even still, it is worth noting that e-cigarettes are not benign products, in addition to their potential for inducing addiction among youth in particular, they can provide considerable exposure to non-negligible levels of many harmful constituents compared with complete nicotine abstinence. For instance, in this study, exclusive e-cigarette users from Poland, who overwhelmingly used refillable tank style devices, had notable urinary concentrations of acrolein, which is a cardiovascular and respiratory toxicant [21,22]. In addition to the potential health risks associated with continued nicotine addiction, the long-term health effects of exposure to acrolein and other cardiovascular toxicants from e-cigarette use have yet to be determined [1]. 

### Strengths and Limitations

This is the first cross-national study to investigate differences in exposure to nicotine, TSNAs, and VOCs in established e-cigarette users, cigarette smokers, and dual users from different countries. An important strength of this study is that we examined exposure levels among a large sample of adults from different international locations with varying levels and types of product use. E-cigarette users in our study used various types of devices. Also, tobacco smokers and dual users smoked a different number of tobacco cigarettes of various brands. By including control subjects from the same populations, we were able to, at least partially, eliminate the bias of environmental and occupational exposures. Other important sources of exposure—such as dietary and environmental exposures, and including potential exposure to secondhand smoke—should be considered in future studies. Sensitivity analyses conducted among non-users across all three countries revealed no statistically significant differences in concentrations of urinary NNAL (*p* > 0.05), which is a tobacco-specific biomarker found in measureable concentrations in secondhand smoke [14]. Because of this, we infer that general background exposure to secondhand smoke was likely similar across countries. Unfortunately, we did not collect detailed information about other sources of exposure in this study. However, our findings were generally consistent with those determined from another study examining similar groups (exclusive e-cigarette users, cigarette smokers, dual users), which did account for potential confounding due to residual exposure from secondhand smoke [3]. 

Our study has several limitations that need to be taken into consideration. First, we did not assess exposure to all potentially harmful compounds that may be present in e-cigarettes emissions. Second, since this was a cross-sectional study, we cannot determine causal relationships between product usage and toxicant exposure. Third, the biomarkers included in this analysis have variable half-lives (mean: 1.5–10 h), [23]. which, in combination with individual metabolic differences, may affect observed biomarker concentrations. Some of the biomarkers measured can also be formed through multiple pathways; for example, exposure to benzyl alcohol in personal care products could form BMA [24]. Fourth, studies have shown significant differences in the metabolism of nicotine and other biomarkers of tobacco exposure according to race/ethnicity [25]. Since the majority of individuals in our study identified as being White, our findings may not entirely translate to tobacco users that identify as members of other racial or ethnic groups. Finally, biological samples were collected across different time windows to accommodate participants’ schedules, which may have introduced some variability across samples. However, the consistency of these findings with those obtained from other similar studies minimizes the extent of this concern [2,3,4,7,18,19,20]. Future studies should seek to build on these data by designing studies better suited to assessing causal relationships between product use and nicotine and toxicant exposure that include individuals from diverse subpopulations of tobacco users.

## 5. Conclusions

Exclusive, long-term e-cigarette use resulted in higher toxicant exposure compared with not using any product, and reduced toxicant exposure compared with cigarette smoking or dual use with cigarettes, while delivering higher levels of nicotine. Differences in exposure to nicotine and toxicants in e-cigarettes from different countries appear to correspond with differences in popularity of various e-cigarette device types across countries. Dual users exhibited a similar level of exposure to nicotine and many toxicants compared with cigarette-only smokers, yet exposure to toxicants in dual users from different countries appears to be correlated with their smoking intensity. 

## Figures and Tables

**Table 1:** Characteristics and pattern of tobacco product use among e-cigarette-only users (*n* = 124), dual users (*n* = 95), and cigarette-only smokers (*n* = 127) from USA, UK, and Poland

	US	UK	Poland	*p*-value
E-Cigarette-Only Users	*n* = 48	*n* = 36	*n* = 40	
Age (years, mean±SD)	40.5 (14.2)	38.5 (11.1)	40.3 (11.8)	0.740
Sex (% (*n*))				
Male	60.4 (29)	80.6 (29) ^c^	47.5 (19) ^b^	0.010
Female	39.6 (19)	19.4 (7) ^c^	52.5 (21) ^b^	
Ethnicity (% (*n*))				
White	93.6 (44)	83.3 (30) ^c^	100 (40) ^b^	0.020
Non-White	6.4 (3)	16.7 (6) ^c^	0 ^b^	
Education (% (*n*))				
Low	50.0 (23)	50.0 (18)	47.5 (19)	0.967
High	50.0 (23)	50.0 (18)	52.5 (21)	
Type of e-cigarette device used (% (*n*))				
Disposable	0 (0)	0 (0)	0 (0)	<0.001
Replaceable cartridge model	4.2 (2)	16.7 (6)	2.5 (1)	
Refillable tank model	85.4 (41) ^b^	36.1 (13) ^a,c^	97.5 (39) ^b^	
Other	10.4 (5) ^b^	47.2 (17) ^a,c^	0 (0) ^b^	
Nicotine concentration in liquid used (% (*n*))				
No nicotine	4.2 (2)	0 (0)	0 (0)	0.141
Very low nicotine (1–4mg)	10.4 (5)	2.9 (1)	0 (0)	
Low nicotine (6–9 mg)	25.0 (12)	20.6 (7)	22.5 (9)	
Medium nicotine (10–15 mg)	27.1 (13)	29.4 (10)	22.5 (9)	
High nicotine (16–24 mg)	27.1 (13)	47.1 (16)	45.0 (18)	
Very high nicotine (>24 mg)	6.3 (3)	0 (0)	10.0 (4)	
Estimated number of puffs per day (mean ± SD)	183.5 (258.1) ^c^	120.4 (81.6)	43.6 (38.4) ^a^	0.003
Dual Users	*n* = 28	*n* = 36	*n* = 31	
Age (years, mean±SD)	38.6 (15.1)	39.3 (13.1)	33.6 (13.2)	0.198
Sex (% (*n*))				
Male	53.6 (15)	69.4 (25)	41.9 (13)	0.075
Female	46.4 (13)	30.6 (11)	58.1 (18)	
Ethnicity (% (*n*))				
White	82.1 (23) ^c^	75.0 (27) ^c^	100 (31) ^a,b^	0.014
Non-White	17.9 (5) ^c^	25.0 (9) ^c^	0 (0) ^a,b^	
Education (% (*n*))				
Low	71.4 (20)	50.0 (18)	56.7 (17)	0.219
High	28.6 (8)	50.0 (18)	43.3 (13)	
Type of e-cigarette device used (% (*n*))				
Disposable	3.6 (1)	19.4 (7) ^c^	0 (0) ^b^	<0.001
Replaceable cartridge model	10.7 (3)	36.1 (13) ^c^	3.2 (1) ^b^	
Refillable tank model	85.7 (24) ^b^	30.6 (11) ^a,c^	96.8 (30) ^b^	
Other	0 (0)	13.9 (5)	0 (0)	
Nicotine concentration in liquid used (% (*n*))				
No nicotine	0 (0)	0 (0)	0 (0)	0.797
Very low nicotine (1–4 mg)	3.6 (1)	4.3 (1)	0 (0)	
Low nicotine (6–9 mg)	17.9 (5)	8.7 (2)	16.1 (5)	
Medium nicotine (10–15 mg)	37.5 (10)	26.1 (6)	41.9 (13)	
High nicotine (16–24 mg)	35.7 (10)	52.2 (12)	38.7 (12)	
Very high nicotine (>24 mg)	7.1 (2)	8.7 (2)	3.2 (1)	
Estimated number of e-cigarette puffs per day (mean ± SD)	65.9 (125.8)	79.5 (75.9)	23.6 (13.2)	0.053
Reported number of cigarettes smoked per day (mean ± SD)	9.4 (6.0)	11.9 (9.6) ^c^	6.8 (6.8) ^b^	0.038
Cigarette-Only Smokers	*n* = 45	*n* = 37	*n* = 45	
Age (years, mean ± SD)	50.1 (11.5) ^b,c^	34.4 (14.0) ^a,c^	43 (11.9) ^a,b^	<0.001
Sex (% (*n*))				
Male	48.9 (22)	56.8 (21)	40.0 (18)	0.316
Female	51.1 (23)	43.2 (16)	60.0 (27)	
Ethnicity (% (*n*))				
White	65.9 (29) ^c^	83.8 (31) ^c^	100 (45) ^a,b^	<0.001
Non-white	34.1 (15) ^c^	16.2 (6) ^c^	0 (0) ^a,b^	
Education (% (*n*))				
Low	62.8 (27)	67.6 (25)	51.1 (23)	0.286
High	37.2 (16)	32.4 (12)	48.9 (22)	
Reported number of cigarettes smoked per day (mean ± SD)	15.7 (11.4)	13.9 (9.0)	16.3 (6.3)	0.469

Note: ^a^ indicates statistically significant difference from the US; ^b^ indicates statistically difference from the UK; ^c^ indicates statistically significant difference from Poland (all *p* < 0.05) Reported *p*-values indicate findings from omnibus statistic; superscript letter notation reflects significant findings adjusted for multiple comparisons (sidak).

**Table 2:** Biomarkers of exposure to nicotine and selected toxicants in urine of exclusive E-cigarette users (*n* = 124) from US, UK and Poland (normalized for creatinine; geometric means, 95% confidence intervals).

Parent Compound	Biomarker	US (*n* = 48)	UK (*n* = 36)	Poland (*n* = 40)	*p*-Value
Nicotine Metabolites (ng/mg creatinine)					
Nicotine	Nicotine Equivalence (nmol/mg)	**35.81 ** ^**c**^ **(24.98–51.33)**	**27.36 ** ^**c**^ **(17.85–41.92)**	**81.34 ** ^**a,b**^ **(54.47–121.45)**	**0.002**
	trans-3′-Hydroxycotinine (HCTT)	**3239.25** **(2208.17–4751.76)**	**2334.34 ** ^**c**^ **(1482.06–3676.73)**	**7273.45 ** ^**a,b**^ **(4747.35–11143.70)**	**0.002**
	Cotinine (COTT)	**1710.08 ** ^**c**^ **(1191.35–2454.66)**	**1331.44 ** ^**c**^ **(867.38–2043.79)**	**3882.26 ** ^**a,b**^ **(2595.98–5805.89)**	**0.002**
	Nicotine (NICT)	**457.22 ** ^**c**^ **(316.74–659.98)**	**422.20 ** ^**c**^ **(273.22–652.41)**	**1205.23 ** ^**a,b**^ **(800.89–1813.69)**	**0.001**
	Cotinine N-oxide (COXT)	**214.18 ** ^**c**^ **(147.90–310.15)**	**150.78 ** ^**c**^ **(97.20–233.87)**	**459.79 ** ^**a,b**^ **(304.46–694.37)**	**0.002**
	Nicotine 1′-oxide (NOXT)	**430.34 ** ^**c**^ **(289.21–640.33)**	**403.70 ** ^**c**^ **(252.01–646.69)**	**1209.91 ** ^**a,b**^ **(777.28–1883.36)**	**0.001**
	Norcotinine (NCTT)	**48.70 ** ^**c**^ **(34.46–68.83)**	**29.64 ** ^**c**^ **(19.66–44.67)**	**94.29 ** ^**a,b**^ **(64.14–138.59)**	**0.001**
	Nornicotine (NNCT)	**27.57 ** ^**c**^ **(20.56–36.98)**	**18.10 ** ^**c**^ **(12.78–25.63)**	**67.00 ** ^**a,b**^ **(48.32–92.89)**	**<0.001**
Minor Tobacco Alkaloids (ng/mg creatinine)					
Anabasine (ANBT)	Anabasine (ANBT)	**1.33 ** ^**c**^ **(0.93–1.89)**	**1.08 ** ^**c**^ **(0.71–1.63)**	**4.66 ** ^**a,b**^ **(3.15–6.87)**	**<0.001**
Anatabine (ANTT)	Anatabine (ANTT)	**0.76 ** ^**c**^ **(0.49–1.15)**	**0.81 ** ^**c**^ **(0.48–1.34)**	**2.46 ** ^**a,b**^ **(1.53–3.94)**	**0.001**
Tobacco-Specific Nitrosamines (TSNAs) (pg/mg creatinine)					
4-methylnitrosamino)-4-(3-pyridyl)-1-butanone (NNK)	4-methylnitrosamino)-4-(3-pyridyl)-1-butanol (NNAL)	**5.14 ^b^** **(3.63–7.26)**	**1.74 ** ^**a,c**^ **(1.15–2.62)**	**4.82 ** ^**b**^ **(3.26–7.12)**	**<0.001**
N’-nitrosoanabasine (NAB)	N’-nitrosoanabasine (NAB)	1.7 (1.35–2.26)	1.17 (0.85–1.58)	1.55 (1.16–2.08)	0.157
N’-nitrosoanatabine (NAT)	N’-nitrosoanatabine (NAT)	3.50 (2.65–4.63)	2.97 2.14–4.13)	4.43 (3.24–6.06)	0.257
Volatile Organic Compounds (VOCs) (ng/mg creatinine)					
Acrolein	N-Acetyl-S-(3-hydroxypropyl)-L-cysteine (3HPMA)	**218.91 ** ^**c**^ **(171.19–279.91)**	**169.18 ** ^**c**^ **(126.40–226.42)**	**436.59 ** ^**a,b**^ **(332.04–574.05)**	**<0.001**
	N-Acetyl-S-(2-carboxyethyl)-L-cysteine (CEMA)	**79.04 ** ^**c**^ **(62.68–99.68)**	**53.45 ** ^**c**^ **(40.59–70.37)**	**156.82 ** ^**a,b**^ **(121.11–203.04)**	**<0.001**
Acrylamide	N-Acetyl-S-(2-carbamoylethyl)-L-cysteine (AAMA)	**47.49 ** ^**b**^ **(37.08–60.81)**	**27.33 ** ^**a**^ **(20.38–36.64)**	**38.46** **(29.20–50.65)**	**0.026**
	N-Acetyl-S-(2-carbamoyl-2-hydroxyethyl)-L-cysteine (GAMA)	**15.17 ** ^**b,c**^ **(12.08–19.04)**	**9.52 ** ^**a**^ **(7.27–12.46)**	**9.62 ** ^**a**^ **(7.46–12.38)**	**0.012**
Acrylonitrile	N-Acetyl-S-(2-cyanoethyl)-L-cysteine (CYMA)	**3.75 ** ^**b**^ **(2.51–5.58)**	**1.38 ** ^**a**^ **(0.85–2.21)**	**2.90** **(1.86–4.52)**	**0.009**
Benzene	N-Acetyl-S-(phenyl)-L-cysteine (PMA)	**0.77 ** ^**c**^ **(0.61–0.95)**	**0.75** **(0.57–0.97)**	**1.14 ** ^**a**^ **(0.89–1.46)**	**0.035**
1,3-Butadiene	N-Acetyl-S-(4-hydroxy-2-buten-1-yl)-L-cysteine (MHBMA3)	**5.22 ** ^**c**^ **(3.97–6.85)**	**4.30 ** ^**c**^ **(3.11–5.93)**	**10.11 ** ^**a,b**^ **(7.46–13.69)**	**<0.001**
	N-Acetyl-S-(3,4-dihydroxybutyl)-L-cysteine (DHBMA)	**206.14 ** ^**c**^ **(170.16–249.72)**	**151.88 ** ^**c**^ **(120.99–190.66)**	**392.56 ** ^**a,b**^ **(317.08–486.01)**	**<0.001**
Crotonaldehyde	N-Acetyl-S-(3-hydroxypropyl-1-methyl)-L-cysteine (HPMMA)	**251.95 ** ^**c**^ **(203.88–311.34)**	**227.82 ** ^**c**^ **(177.26–292.81)**	**421.77 ** ^**a,b**^ **(333.21–533.85)**	**0.002**
Cyanide	2-Aminothiazoline-4-carboxylic acid (ATCA)	**67.46 ** ^**c**^ **(50.60–89.92)**	**66.54 ** ^**c**^ **(47.32–93.56)**	**121.85 ** ^**a,b**^ **(88.48–167.81)**	**0.017**
N,N-Dimethylformamide; methyl isocyanate	N-Acetyl-S-(N-methylcarbamoyl)-L-cysteine (AMCC)	**160.50 ** ^**b**^ **(128.50–200.45)**	**61.50 ** ^**a,c**^ **(47.24–80.04)**	**224.10 ** ^**b**^ **(174.96–287.03)**	**<0.001**
Ethylene oxide; acrylonitrile; vinyl chloride	N-Acetyl-S-(2-hydroxyethyl)-L-cysteine (HEMA) ***	0.55 (0.44–0.69)	0.44 (0.33–0.57)	0.50 (0.39–0.64)	0.442
Propylene oxide	N-Acetyl-S-(2-hydroxypropyl)-L-cysteine (2HPMA)	39.34 (30.11–51.41)	31.51 (22.94–43.26)	40.75 (30.25–54.89)	0.491
Styrene	Mandelic acid (MA)	98.37 (79.67–121.45)	102.37 (79.73–131.43)	134.56 (106.41–170.15)	0.144
Styrene; ethylbenzene	Phenylglyoxylic acid (PGA)	**100.57 ** ^**c**^ **(79.01–128.01)**	**72.03 ** ^**c**^ **(54.11–95.88)**	**160.44 ** ^**a,b**^ **(122.64–209.88)**	**0.001**
Toluene; benzyl alcohol	N-Acetyl-S-(benzyl)-L-cysteine (BMA)	4.14 (3.18–5.36)	4.84 (3.55–6.61)	5.78 (4.31–7.74)	0.266
Xylene	2-Methylhippuric acid (2MHA)	17.66 (13.49–23.11)	10.75 (7.81–14.78)	16.73 (12.40–22.58)	0.063
	3- + 4-Methylhippuric acids (34MHA)	**134.38 ** ^**b**^ **(103.81–173.94)**	**50.29 ** ^**a,c**^ **(37.03–68.29)**	**97.28 ** ^**b**^ **(72.98–129.66)**	**<0.001**

Note: Bolded values denote statistically significant differences in geometric mean concentrations between at least two of the countries. ^a^ indicates statistically significant difference from the US; ^b^ indicates statistically difference from the UK; ^c^ indicates statistically significant difference from Poland (all *p* < 0.05). Reported *p*-values indicate findings from omnibus statistic; superscript letter notation reflects significant findings adjusted for multiple comparisons (sidak). Geometric means are adjusted for urinary creatinine, age, sex, and race. ***: These analytes had more than 40% of measured values fall below the analytical limit of detection (LOD).

**Table 3:** Biomarkers of exposure to nicotine and selected toxicants in urine of dual users (*n* = 95) from USA, UK, and Poland (normalized for creatinine; geometric means, 95% confidence intervals).

Parent Compound	Biomarker	US (*n* = 28)	UK (*n* = 36)	Poland (*n* = 31)	*p*-Value
Nicotine Metabolites (ng/mg Creatinine)					
Nicotine	Nicotine Equivalence (nmol/mg)	**22.36 ** ^**c**^ **(14.13–35.37)**	**32.07** **(21.04–48.89)**	**53.83 ** ^**a**^ **(33.85–85.56)**	**0.045**
	trans-3′-Hydroxycotinine (HCTT)	2049.55 (1248.36–3364.91)	2163.15 (1371.44–3411.88)	4584.93 (2776.80–7570.42)	0.068
	Cotinine (COTT)	**967.48 ** ^**c**^ **(597.39–1566.82)**	**1485.78** **(953.90–2314.22) **	**2659.48 ** ^**a**^ **(1633.10–4330.89)**	**0.025**
	Nicotine (NICT)	**272.30 ** ^**b,c**^ **(163.90–452.37)**	**661.17 ** ^**a**^ **(414.65–1054.23)**	**753.84 ** ^**a**^ **(451.13–1259.68)**	**0.014**
	Cotinine N-oxide (COXT)	144.81 (90.74–231.09)	155.62 (101.27–239.14)	286.30 (178.44–459.33)	0.121
	Nicotine 1′-oxide (NOXT)	**253.11 ** ^**c**^ **(145.89–439.11)**	**617.51** **(372.15–1024.62)**	**781.42 ** ^**a**^ **(447.58–1364.25)**	**0.017**
	Norcotinine (NCTT)	32.57 (20.51–51.71)	46.78 (30.58–71.55)	69.68 (43.64–111.23)	0.099
	Nornicotine (NNCT)	**20.96 ** ^**c**^ **(13.98–31.43)**	**34.17** **(23.55–49.58)**	**51.26 ** ^**a**^ **(34.03–77.21)**	**0.016**
Minor Tobacco Alkaloids (ng/mg creatinine)					
Anabasine (ANBT)	Anabasine (ANBT)	2.12 (1.33–3.35)	4.27 (2.79–6.52)	2.86 (1.79–4.56)	0.105
Anatabine (ANTT)	Anatabine (ANTT)	2.66 (1.54–4.56)	5.90 (3.59-9.69)	2.42 (1.40-4.18)	0.051
Tobacco-Specific Nitrosamines (TSNAs) (pg/mg creatinine)					
4-methylnitrosamino)-4-(3-pyridyl)-1-butanone (NNK)	4-methylnitrosamino)-4-(3-pyridyl)-1-butanol (NNAL)	47.39 (30.00–74.85)	55.80 (36.65–84.94)	30.99 (19.51–49.20)	0.227
N’-nitrosoanabasine (NAB)	N’-nitrosoanabasine (NAB)	**3.72** **(2.36–5.85)**	**6.59 ** ^**c**^ **(4.33–10.00)**	**2.52 ** ^**b**^ **(1.59–3.99)**	**0.021**
N’-nitrosoanatabine (NAT)	N’-nitrosoanatabine (NAT)	**14.53 ** **(8.54–24.72)**	**29.53 ** ^**c**^ **(18.11–48.13)**	**11.04 ** ^**b**^ **(6.45–18.91)**	**0.040**
Volatile Organic Compounds (VOCs) (ng/mg creatinine)					
Acrolein	N-Acetyl-S-(3-hydroxypropyl)-L-cysteine (3HPMA)	**285.36 ** ^**b**^ **(197.79–411.67)**	**574.63 ** ^**a**^ **(410.29–804.78)**	**341.92** **(236.01–495.35)**	**0.025**
	N-Acetyl-S-(2-carboxyethyl)-L-cysteine (CEMA)	87.25 (63.61–119.66)	141.75 (106.02–189.51)	130.57 (94.85-179.73)	0.079
Acrylamide	N-Acetyl-S-(2-carbamoylethyl)-L-cysteine (AAMA)	57.08 (41.69–78.13)	85.33 (63.93–113.88)	51.96 (37.81–71.39)	0.079
	N-Acetyl-S-(2-carbamoyl-2-hydroxyethyl)-L-cysteine (GAMA)	**16.62 ** **(12.56–21.99)**	**26.02 ** ^**c**^ **(20.11–33.66)**	**12.93 ** ^**b**^ **(9.74–17.17)**	**0.004**
Acrylonitrile	N-Acetyl-S-(2-cyanoethyl)-L-cysteine (CYMA)	36.35 (21.84–60.48)	51.38 (32.17–82.05)	24.93 (14.89-41.73)	0.176
Benzene	N-Acetyl-S-(phenyl)-L-cysteine (PMA)	**0.55 ** ^**b**^ **(0.42–0.73)**	**1.48 ** ^**a,c**^ **(1.15–1.89)**	**0.80 ** ^**b**^ **(0.60–1.05)**	**<0.001**
1,3-Butadiene	N-Acetyl-S-(4-hydroxy-2-buten-1-yl)-L-cysteine (MHBMA3)	**15.31 ** ^**b**^ **(10.23–22.89)**	**35.20 ** ^**a,c**^ **(24.31–50.96)**	**15.14 ** ^**b**^ **(10.07–22.74)**	**0.006**
	N-Acetyl-S-(3,4-dihydroxybutyl)-L-cysteine (DHBMA)	**165.84 ** ^**b,c**^ **(128.5–214.02)**	**298.47 ** ^**a**^ **(236.09–377.31)**	**271.85 ** ^**a**^ **(210.04–351.86)**	**0.004**
Crotonaldehyde	N-Acetyl-S-(3-hydroxypropyl-1-methyl)-L-cysteine (HPMMA)	**464.34 ** ^**b**^ **(318.75–676.42)**	**1211.53 ** ^**a,c**^ **(857.35–1712.01)**	**443.66 ** ^**b**^ **(303.24–649.09)**	**0.004**
Cyanide	2-Aminothiazoline-4-carboxylic acid (ATCA)	**57.43 ** ^**b**^ **(43.73–75.42)**	**144.93 ** ^**a,c**^ **(112.82–186.18)**	**81.21 ** ^**b**^ **(61.65–106.98)**	**<0.001**
N,N-Dimethylformamide; methyl isocyanate	N-Acetyl-S-(N-methylcarbamoyl)-L-cysteine (AMCC)	166.42 (122.04–226.93)	177.78 (133.68–236.42)	219.07 (160.08–299.79)	0.486
Ethylene oxide; acrylonitrile; vinyl chloride	N-Acetyl-S-(2-hydroxyethyl)-L-cysteine (HEMA) ***	0.85 (0.58–1.23)	1.18 (0.83–1.66)	0.79 (0.54–1.16)	0.302
Propylene oxide	N-Acetyl-S-(2-hydroxypropyl)-L-cysteine (2HPMA)	45.65 (32.66–63.78)	69.28 (50.94–94.22)	44.38 (31.64–62.25)	0.128
Styrene	Mandelic acid (MA)	**129.37 ** ^**b**^ **(97.75–171.21)**	**228.04 ** ^**a**^ **(176.26–295.02)**	**157.62 ** **(118.72–209.27)**	**0.019**
Styrene, ethylbenzene	Phenylglyoxylic acid (PGA)	100.85 (71.14–142.97)	128.60 (93.31–177.24)	124.89 (87.74–177.75)	0.575
Toluene; benzyl alcohol	N-Acetyl-S-(benzyl)-L-cysteine (BMA)	**2.75 ** ^**b,c**^ **(1.97–3.82)**	**6.22 ** ^**a**^ **(4.59–8.43)**	**5.11 ** ^**a**^ **(3.65–7.13)**	**0.002**
Xylene	2-Methylhippuric acid (2MHA)	**30.77** **(21.13–44.79)**	**56.78 ** ^**c**^ **(40.20–80.18)**	**21.11 ** ^**b**^ **(14.44–30.87)**	**0.003**
	3- + 4-Methylhippuric acids (34MHA)	**206.80** **(141.11–303.06)**	**269.53 ** ^**c**^ **(189.69–382.97)**	**121.17 ** ^**b**^ **(82.32–178.35)**	**0.024**

Note: Bolded values denote statistically significant differences in geometric mean concentrations between at least two of the countries. ^a^ indicates statistically significant difference from the US; ^b^ indicates statistically difference from the UK; ^c^ indicates statistically significant difference from Poland (all *p* < 0.05). Reported *p*-values indicate findings from omnibus statistic; superscript letter notation reflects significant findings adjusted for multiple comparisons (sidak). Geometric means are adjusted for urinary creatinine, age, sex, and race. ***: These analytes had more than 40% of measured values fall below the analytical limit of detection (LOD).

**Table 4:** Biomarkers of exposure to nicotine and selected toxicants in urine of exclusive smokers (*n* = 127) from USA, UK, and Poland (normalized for creatinine; geometric means, 95% confidence intervals).

Parent Compound	Biomarker	USA (*n* = 45)	UK (*n* = 37)	Poland (*n* = 45)	*p*-Value
Nicotine Metabolites (ng/mg creatinine)					
Nicotine	Nicotine Equivalence (nmol/mg)	27.36 (19.76–37.88)	30.86 (21.77–43.74)	38.53 (28.35–52.35)	0.348
	Trans-3′-Hydroxycotinine (HCTT)	2103.85 (1435.37–3083.66)	2402.85 (1595.02–3619.83)	2970.19 (2071.79–4258.16)	0.465
	Cotinine (COTT)	1294.10 (930.73–1799.32)	1451.28 (1019.39–2066.13)	1678.40 (1230.38–2289.55)	0.563
	Nicotine (NICT)	**236.74 ** ^**c**^ **(174.07–399.59)**	**433.25 ** **(277.55–676.27)**	**678.44 ** ^a^ **(458.68–1003.49)**	**0.011**
	Cotinine *n*-oxide (COXT)	159.10 (113.78–222.47)	165.80 (115.75–237.47)	199.44 (145.42–273.50)	0.615
	Nicotine 1′-oxide (NOXT)	**261.05 ** ^c^ **(170.01–400.81)**	**440.11** **(277.95–696.85)**	**655.24 ** ^**a**^ **(437.48–981.39)**	**0.018**
	Norcotinine (NCTT)	42.25 (31.02–57.54)	45.79 (32.88–63.75)	53.72 (40.16–71.86)	0.558
	Nornicotine (NNCT)	**25.00 ** ^**c**^ **(18.34–34.08)**	**30.03 ** **(21.54–41.86)**	**47.62 ** ^**a**^ **(35.56–63.75)**	**0.015**
Minor Tobacco Alkaloids (ng/mg creatinine)					
Anabasine (ANBT)	Anabasine (ANBT)	2.95 (2.16–4.01)	3.78 (2.71–5.25)	4.78 (3.57–6.36)	0.110
Anatabine (ANTT)	Anatabine (ANTT)	**2.97 ** ^**c**^ **(2.08–4.23)**	**5.71** **(3.90–8.34)**	**6.62 ** ^**a**^ **(4.74–9.23)**	**0.009**
Tobacco-Specific Nitrosamines (TSNAs) (pg/mg creatinine)					
4-methylnitrosamino)-4-(3-pyridyl)-1-butanone (NNK)	4-methylnitrosamino)-4-(3-pyridyl)-1-butanol (NNAL)	87.78 (66.94–115.08)	94.38 (70.60–126.18)	85.12 (65.95–109.87)	0.878
N’-nitrosoanabasine (NAB)	N’-nitrosoanabasine (NAB)	8.38 (6.16–11.41)	8.15 (5.86–11.34)	8.94 (6.68–11.95)	0.916
N’-nitrosoanatabine (NAT)	N’-nitrosoanatabine (NAT)	26.57 (18.46–38.24)	38.73 (26.22–57.23)	46.29 (32.84–65.23)	0.124
Volatile Organic Compounds (VOCs) (ng/mg creatinine)					
Acrolein	N-Acetyl-S-(3-hydroxypropyl)-L-cysteine (3HPMA)	**241.90 ** ^**b,c**^ **(186.56–313.65)**	**602.98 ** ^**a**^ **(456.46–796.52)**	**584.57 ** ^**a**^ **(457.67–746.64)**	**<0.001**
	N-Acetyl-S-(2-carboxyethyl)-L-cysteine (CEMA)	**90.03 ** ^**b,c**^ **(71.77–112.92)**	**160.03 ** ^**a**^ **(125.53–204.02)**	**154.84 ** ^**a**^ **(125.07–191.68)**	**0.002**
Acrylamide	N-Acetyl-S-(2-carbamoylethyl)-L-cysteine (AAMA)	**54.18 ** ^**c**^ **(43.74–67.12)**	**79.91** **(63.52–100.53)**	**88.90 ** ^**a**^ **(72.66–108.78)**	**0.008**
	N-Acetyl-S-(2-carbamoyl-2-hydroxyethyl)-L-cysteine (GAMA)	**19.22** **(15.52–23.80)**	**22.12** **(17.59–27.80)**	**20.25** **(16.55–24.76)**	**0.714**
Acrylonitrile	N-Acetyl-S-(2-cyanoethyl)-L-cysteine (CYMA)	**43.66 ** ^**c**^ **(32.55–58.55)**	**66.45** **(48.51–91.00)**	**88.88 ** ^**a**^ **(67.14–117.19)**	**0.006**
Benzene	N-Acetyl-S-(phenyl)-L-cysteine (PMA)	0.66 (0.50–0.86)	0.78 (0.58–1.03)	0.74 (0.57–0.94)	0.740
1,3-Butadiene	N-Acetyl-S-(4-hydroxy-2-buten-1-yl)-L-cysteine (MHBMA3)	**13.50 ** ^**b,c**^ **(9.97–18.28)**	**38.70 ** ^**a**^ **(27.96–53.55)**	**29.08 ** ^**a**^ **(21.85–38.69)**	**0.001**
	N-Acetyl-S-(3,4-dihydroxybutyl)-L-cysteine (DHBMA)	**163.89 ** ^**b,c**^ **(134.44–199.79)**	**250.76 ** ^**a**^ **(202.80–310.05)**	**273.71 ** ^**a**^ **(227.12–329.86)**	**0.002**
Crotonaldehyde	N-Acetyl-S-(3-hydroxypropyl-1-methyl)-L-cysteine (HPMMA)	**352.03 ** ^**b,c**^ **(265.92–466.04)**	**1006.22 ** ^**a**^ **(744.94–1359.13)**	**888.93 ** ^**a**^ **(682.47–1157.83)**	**<0.001**
Cyanide	2-Aminothiazoline-4-carboxylic acid (ATCA)	83.59 (64.98–107.54)	112.84 (86.14–147.81)	102.90 (81.16–130.46)	0.318
N,N-Dimethylformamide; methyl isocyanate	N-Acetyl-S-(N-methylcarbamoyl)-L-cysteine (AMCC)	173.99 (136.45–221.85)	214.47 (165.30–278.28)	264.52 (210.40–332.56)	0.070
Ethylene oxide; acrylonitrile; vinyl chloride	N-Acetyl-S-(2-hydroxyethyl)-L-cysteine (HEMA) ***	0.86 (0.67–1.11)	1.02 (0.77–1.34)	1.18 (0.92–1.49)	0.255
Propylene oxide	N-Acetyl-S-(2-hydroxypropyl)-L-cysteine (2HPMA)	49.33 (38.21–63.68)	50.40 (38.33–66.26)	54.85 (43.12–69.77)	0.833
Styrene	Mandelic acid (MA)	**125.58 ** ^**b**^ **(102.80–153.40)**	**234.14 ** ^**a,c**^ **(188.94–290.15)**	**161.59 ** ^b^ **(133.82–195.11)**	**0.001**
Styrene; ethylbenzene	Phenylglyoxylic acid (PGA)	**82.13 ** ^**c**^ **(61.16–110.28)**	**110.46** **(80.54–151.50)**	**153.52 ** ^**a**^ **(116.29–202.65)**	**0.018**
Toluene; benzyl alcohol	N-Acetyl-S-(benzyl)-L-cysteine (BMA)	3.88 (2.94–5.11)	4.59 (3.42–6.18)	4.73 (3.64–6.13)	0.599
Xylene	2-Methylhippuric acid (2MHA)	37.26 (29.09–47.71)	53.32 (40.90–69.50)	35.37 (28.01–44.65)	0.072
	3- + 4-Methylhippuric acids (34MHA)	233.85 (174.66–313.08)	339.90 (248.62–464.69)	198.75 (150.98–261.63)	0.052

Note: Bolded values denote statistically significant differences in geometric mean concentrations between at least two of the countries. ^a^ indicates statistically significant difference from the US; ^b^ indicates statistically difference from the UK; ^c^ indicates statistically significant difference from Poland (all *p* < 0.05). Reported *p*-values indicate findings from omnibus statistic; superscript letter notation reflects significant findings adjusted for multiple comparisons (sidak). Geometric means are adjusted for urinary creatinine, age, sex, and race.

## Supplementary Materials

The following are available online at https://www.mdpi.com/2305-6304/8/4/88/s1, Table S1: Analytic Limits of Detection (LODs) for biomarkers of exposure to nicotine, tobacco-specific nitrosamines (TSNAs), and volatile organic compounds (VOCs); Table S2: Biomarkers of exposure to nicotine and minor tobacco alkaloids in urine of e-cigarette users, tobacco smokers, dual users, and non-users (normalized for creatinine; adjusted geometric means, 95% confidence intervals); Table S3: Biomarkers of exposure to tobacco specific nitrosamines (TSNAs) in urine of e-cigarette users, tobacco smokers, dual users, and non-users (normalized for creatinine; adjusted geometric means, 95% confidence intervals); Table S4: Biomarkers of exposure from volatile organic compounds (VOCs) in urine of e-cigarette users, tobacco smokers, dual users, and non-users (normalized for creatinine; adjusted geometric means, 95% confidence intervals); Table S5: Biomarkers of exposure to nicotine and selected toxicants in urine of Exclusive E-cigarette Users (*n* = 124) from USA, UK and Poland (normalized for creatinine; geometric means, 95% confidence intervals) who used different types of vaping devices.
