# Supplementary material for: Differences in Exposure to Nicotine, Tobacco-Specific Nitrosamines, and Volatile Organic Compounds among Electronic Cigarette Users, Tobacco Smokers, and Dual Users from Three Countries
Source: Toxics. 2020 Oct 14;8(4):88. doi: 10.3390/toxics8040088 (PMC7712026; doi:10.3390/toxics8040088)
Supplement: Supplementary file 1 [file toxics-08-00088-s001.pdf]

Differences in Exposure to Nicotine, Tobacco-Specific Nitrosamines, and Volatile Organic Compounds among Electronic Cigarette Users, Tobacco Smokers, and Dual Users from Three Countries

Danielle M. Smith, Lion Shahab, Benjamin C. Blount, Michal Gawron, Leon Kosminder, Andrzej Sobczak, Baoyun Xia, Connie S. Sosnoff, and Maciej L. Goniewicz

Table S1: Analytic Limits of Detection (LODs) for biomarkers of exposure to nicotine, tobacco-specific nitrosamines (TSNAs), and volatile organic compounds (VOCs).

Parent Compound	Biomarker	Limit of detection (LOD) ng/mL
	Nicotine Metabolites	
Nicotine	Nicotine Equivalence (nmol/mg)	----
	trans-3′-Hydroxycotinine (HCTT)	0.030
	Cotinine (COTT)	0.030
	Nicotine (NICT)	10.5
	Cotinine N-oxide (COXT)	2.02
	Nicotine 1′-oxide (NOXT)	2.5
	Norcotinine (NCTT)	1.11
	Nornicotine (NNCT)	2.5
Anabasine (ANBT)	Anabasine (ANBT)	0.51
Anatabine (ANTT)	Anatabine (ANTT)	0.39
	Tobacco Specific Nitrosamines (TSNAs)	
4-methylnitrosamino)-4-(3-pyridyl)-1-butanone (NNK)	4-methylnitrosamino)-4-(3-pyridyl)-1-butanol (NNAL)	0.0006
N′-nitrosoanabasine (NAB)	N′-nitrosoanabasine (NAB)	0.0016
N′-nitrosoanatabine (NAT)	N′-nitrosoanatabine (NAT)	0.004
	Volatile Organic Compounds (VOCs)	
Acrolein	N-Acetyl-S-(3-hydroxypropyl)-L-cysteine (3HPMA)	13.0
	N-Acetyl-S-(2-carboxyethyl)-L-cysteine (CEMA)	8.0
	N-Acetyl-S-(2-carbamoylethyl)-L-cysteine (AAMA)	2.2

Acrylamide	N-Acetyl-S-(2-carbamoyl-2-hydroxyethyl)-L-cysteine (GAMA)	9.4
Acrylonitrile	N-Acetyl-S-(2-cyanoethyl)-L-cysteine (CYMA)	0.5
Benzene	N-Acetyl-S-(phenyl)-L-cysteine (PMA)	0.6
1,3-Butadiene	N-Acetyl-S-(4-hydroxy-2-buten-1-yl)-L-cysteine (MHBMA3)	0.6
	N-Acetyl-S-(3,4-dihydroxybutyl)-L-cysteine (DHBMA)	5.0
Crotonaldehyde	N-Acetyl-S-(3-hydroxypropyl-1-methyl)-L-cysteine (HPMMA)	2.0
Cyanide	2-Aminothiazoline-4-carboxylic acid (ATCA)	5.5
N,N-Dimethylformamide	N-Acetyl-S-(N-methylcarbamoyl)-L-cysteine (AMCC)	5.5
Ethylene oxide, acrylonitrile, vinyl chloride	N-Acetyl-S-(2-hydroxyethyl)-L-cysteine (HEMA)***	0.6
Propylene oxide	N-Acetyl-S-(2-hydroxypropyl)-L-cysteine (2HPMA)	1.3
Styrene	Mandelic acid (MA)	12.0
Styrene, ethylbenzene	Phenylglyoxylic acid (PGA)	12.0
Toluene	N-Acetyl-S-(benzyl)-L-cysteine (BMA)	0.5
Xylene	2-Methylhippuric acid (2MHA)	5.0
	3- + 4-Methylhippuric acids (34MHA)	8.0

Table S2. Biomarkers of exposure to nicotine and minor tobacco alkaloids in urine of e-cigarette users, tobacco smokers, dual users, and non-users (normalized for creatinine; adjusted geometric means, 95% confidence intervals).

Parent Compound	Biomarker	Non-users (a) (n=110)	E-cigarette users (b) (n=124)	Dual users (c) (n=95)	Cigarette smokers (d) (n=127)
	Nicotine Metabolites (ng/mg creatinine)				
Nicotine	Nicotine Equivalence (nmol/mg)	0.11 (0.09–0.14)[bcd]	45.1 (36.6–55.4)[ad]	37.4 (29.6–47.2)[a]	29.7 (24.2–36.3)[ab]
	trans-3′-Hydroxycotinine (HCTT)	1.5 (1.1–1.9)[bcd]	34,029 (3171–5117)[ad]	23,076 (2346–4034)[a]	2,275 (1798–2879)[ab]
	Cotinine (COTT)	1.0 (0.8–1.3)[bcd]	2,130 (1700–2669)[ad]	1,746 (1353–2253)[a]	1,376 (1103–1719)[ab]
	Nicotine (NICT)	8.42 (6.62–10.70)[bcd]	638 (508–800)[ad]	570 (441–737)[a]	393 (314–491)[ab]
	Cotinine N-oxide (COXT)	1.57 (1.26–1.96)[bcd]	257.67 (209.3–317.1)[ad]	1202.7 (160.2–256.4)[a]	1162.9 (132.8–199.9)[ab]
	Nicotine 1′-oxide (NOXT)	2.07 (1.60–2.67)[bcd]	616 (484–786)[ad]	551 (418–725)[a]	387 (305–491)[ab]
	Norcotinine (NCTT)	0.84 (0.68–1.04)[bcd]	554.0 (44.2–65.7)[a]	52.5 (41.9–65.7)[a]	43.8 (36.0–53.2)[a]
	Nornicotine (NNCT)	1.87 (1.53–2.26)[bcd]	33.8 (28.1–40.5)[a]	36.6 (29.7–45.1)[a]	31.0 (25.8–37.1)[a]
	Minor Tobacco Alkaloids (ng/mg creatinine)				
Anabasine (ANBT)	Anabasine (ANBT)	0.39 (0.31–0.48)[bcd]	1.96 (1.60–2.40)[acd]	3.30 (2.62–4.14)[ab]	3.46 (2.83–4.22)[ab]
Anatabine (ANTT)	Anatabine (ANTT)	0.31 (0.24–0.38)[bcd]	1.17 (0.92–1.47)[acd]	3.71 (2.85–4.83)[ab]	4.38 (3.48–5.50)[ab]

*Superscript letters denote statistically significant differences between study groups (sidak adjusted p-value < 0.05). Geometric means are adjusted for age, sex, race, and country of residence.

Table S3. Biomarkers of exposure to tobacco specific nitrosamines (TSNAs) in urine of e-cigarette users, tobacco smokers, dual users, and non-users (normalized for creatinine; adjusted geometric means, 95% confidence intervals).

Parent Compound	Biomarker	Non-users (a) (*n* = 110)	E-cigarette users (b) (*n* = 124)	Dual users (c) (*n* = 95)	Cigarette smokers (d) (*n* = 127)
Tobacco-Specific Nitrosamines (TSNAs) (pg/mg creatinine)					
4-methylnitrosamino)-4-(3-pyridyl)-1-butanone (NNK)	4-methylnitrosamino)-4-(3-pyridyl)-1-butanol (NNAL)	1.05 (0.84–1.30)^{bcd}	3.71 (3.02–4.57)^{acd}	48.5 (38.4–61.2)^{abd}	82.9 (67.7–101.4)^{abc}
N′-nitrosoanabasine (NAB)	N′-nitrosoanabasine (NAB)	1.29 (1.06–1.55)^{cd}	1.49 (1.25–1.79)^{cd}	4.32 (3.53–5.28)^{abd}	8.11 (6.81–9.64)^{abc}
N′-nitrosoanatabine (NAT)	N′-nitrosoanatabine (NAT)	3.16 (2.55–3.91)^{cd}	3.63 (2.96–4.44)^{cd}	18.38 (14.64–23.08)^{abd}	34.00 (27.90–41.44)^{abc}

*Superscript letters denote statistically significant differences between study groups (sidak adjusted *p*-value <0.05). Geometric means are adjusted for age, sex, race, and country of residence.

Table S4. Biomarkers of exposure from volatile organic compounds (VOCs) in urine of e-cigarette users, tobacco smokers, dual users, and non-users (normalized for creatinine; adjusted geometric means, 95% confidence intervals).

Parent Compound	Biomarker	Non-users (a) (*n* = 110)	E-cigarette users (b) (*n* = 124)	Dual users (c) (*n* = 95)	Cigarette smokers (d) (*n* = 127)
Volatile Organic Compounds (VOCs) (ng/mg creatinine)					
Acrolein	N-Acetyl-S-(3-hydroxypropyl)-L-cysteine (3HPMA)	282 (235–338)[cd]	257 (216–304)[cd]	405 (334–492)[ab]	414 (350–489)[ab]
	N-Acetyl-S-(2-carboxyethyl)-L-cysteine (CEMA)	99.1 (84.7–115.9)	90.6 (78.1–105.0)[cd]	126.9 (107.3–150.1)[ab]	122.6 (105.9–141.8)[b]
Acrylamide	N-Acetyl-S-(2-carbamoylethyl)-L-cysteine (AAMA)	33.7 (28.8–39.3)[cd]	37.9 (32.7–44.0)[cd]	66.4 (56.2–78.4)[ab]	71.0 (61.4–82.1)[ab]
	N-Acetyl-S-(2-carbamoyl-2-hydroxyethyl)-L-cysteine (GAMA)	11.7 (10.1–13.5)[cd]	11.4 (9.9–13.1)[cd]	18.7 (16.0–21.8)[ab]	19.9 (17.4–22.8)[ab]
Acrylonitrile	N-Acetyl-S-(2-cyanoethyl)-L-cysteine (CYMA)	1.39 (1.12–1.74)[bcd]	2.57 (2.08–3.16)[acd]	39.14 (30.91–49.57)[abd]	61.08 (49.74–74.98)[abc]
Benzene	N-Acetyl-S-(phenyl)-L-cysteine (PMA)	0.86 (0.74–1.00)	0.87 (0.75–1.01)	0.92 (0.78–1.08)	0.70 (0.60–0.80)
1,3-Butadiene	N-Acetyl-S-(4-hydroxy-2-buten-1-yl)-L-cysteine (MHBMA3)	7.28 (6.03–8.79)[cd]	6.20 (5.18–7.40)[cd]	21.17 (17.31–25.88)[ab]	23.10 (19.40–27.51)[ab]
	N-Acetyl-S-(3,4-dihydroxybutyl)-L-cysteine (DHBMA)	249 (220–283)	240 (212–270)[cd]	251 (220–288)	216 (191–243)
Crotonaldehyde	N-Acetyl-S-(3-hydroxypropyl-1-methyl)-L-cysteine (HPMMA)	362 (305–431)[cd]	293 (249–344)[cd]	665 (554–799)[ab]	633 (540–742)[ab]
Cyanide	2-Aminothiazoline-4-carboxylic acid (ATCA)	104.7 (88.3–124.3)	89.0 (75.7–104.5)	95.5 (79.5–114.6)	93.3 (79.6–109.4)
N,N-Dimethylformamide; methyl isocyanate	N-Acetyl-S-(N-methylcarbamoyl)-L-cysteine (AMCC)	97 (83–114)[bcd]	139 (119–161)[acd]	206 (174–243)[ab]	204 (176–236)[ab]
Ethylene oxide; acrylonitrile; vinyl chloride	N-Acetyl-S-(2-hydroxyethyl)-L-cysteine (HEMA)***	0.67 (0.57–0.79)[cd]	0.51 (0.44–0.60)[cd]	0.99 (0.83–1.17)[ab]	0.97 (0.84–1.13)[ab]
Propylene oxide	N-Acetyl-S-(2-hydroxypropyl)-L-cysteine (2HPMA)	34.1 (28.5–40.8)[cd]	37.8 (31.9–44.7)[c]	55.5 (45.9–67.2)[ab]	50.0 (42.3–59.0)[a]

Styrene	Mandelic acid (MA)	130 (113–149)[c]	111 (98–127)[cd]	175 (151–203)[ab]	158 (139–180)[b]
Styrene, ethylbenzene	Phenylglyoxylic acid (PGA)	103 (91–126)	111 (94–130)	125 (103–150)	108 (91–126)
Toluene; benzyl alcohol	N-Acetyl-S-(benzyl)-L-cysteine (BMA)	8.20 (6.89–9.75)[bcd]	5.25 (4.45–6.18)[a]	4.72 (3.92–5.68)[a]	4.09 (3.48–4.80)[a]
Xylene	2-Methylhippuric acid (2MHA)	17.2 (14.4–20.5)[cd]	14.8 (12.5–17.5)[cd]	36.2 (29.9–43.7)[ab]	39.0 (33.0–45.9)[ab]
	3- + 4-Methylhippuric acids (34MHA)	88.6 (74.0–105.9)[cd]	89.2 (75.3–105.6)[cd]	206.3 (170.4–249.8)[ab]	236.9 (200.6–279.7)[ab]

*Superscript letters denote statistically significant differences between study groups (sidak adjusted *p*-value < 0.05). Geometric means are adjusted for age, sex, race, and country of residence.

Table S5. Biomarkers of exposure to nicotine and selected toxicants in urine of Exclusive E-cigarette Users (n=124) from USA, UK and Poland (normalized for creatinine; geometric means, 95% confidence intervals) who used different types of vaping devices.

**Parent Compound**	**Biomarker**	**Replaceable cartridge model (*n* = 9)**	**Refillable tank model (*n* = 93)**	**Other (*n* = 22)**	***p*-value**
Nicotine	Nicotine Equivalence (nmol/mg)	28.35 (12.40–64.77)	37.75 (28.95–49.22)[c]	90.36 (50.15–162.79)[b]	0.023
	trans-3′-Hydroxycotinine (HCTT)	2101.6 (878.1–5029.5)[c]	3324.9 (2512.3–4400.1)[c]	8827.4 (4740.2–16438.3) [a,b]	0.010
	Cotinine (COTT)	1396.4 (608.8–3202.7)	1807.9 (1384.8–2360.0)[c]	4340.8 (2402.7–7842.1)[b]	0.026
	Nicotine (NICT)	570.5 (243.1–1338.8)	528.9 (402.1–695.5)	1161.6 (632.5–2133.0)	0.102
	Cotinine N-oxide (COXT)	154.24 (65.84–361.32)	218.49 (166.23–287.17)	508.98 (277.51–933.48)	0.030
	Nicotine 1′-oxide (NOXT)	489.65 (194.49–1232.7)	511.13 (379.99–687.51)	1172.94 (607.53–2264.5)	0.099
	Norcotinine (NCTT)	32.89 (14.92–72.51)[c]	45.74 (35.48–58.95)[c]	109.66 (62.43–192.59)[a,b]	0.015
	Nornicotine (NNCT)	24.00 (12.22–47.11)	29.06 (23.40–36.09)[c]	59.11 (36.56–95.57)[b]	0.028
Anabasine (ANBT)	Anabasine (ANBT)	3.02 (1.32–6.86)	1.78 (1.36–2.32)	1.93 (1.07–3.47)	0.529

Anatabine (ANTT)	Anatabine (ANTT)	3.14 (1.17–8.42)	1.03 (0.74–1.41)	1.12 (0.55–2.25)	0.131
4-methylnitrosamino)-4-(3-pyridyl)-1-butanone (NNK)	4-methylnitrosamino)-4-(3-pyridyl)-1-butanol (NNAL)	4.99 (2.20–11.26)	3.33 (2.56–4.34)	4.72 (2.64–8.42)	0.521
N′-nitrosoanabasine (NAB)	N′-nitrosoanabasine (NAB)	2.52 (1.37–4.60)	1.40 (1.14–1.70)	1.60 (1.03–2.45)	0.236
N′-nitrosoanatabine (NAT)	N′-nitrosoanatabine (NAT)	8.08 (4.26–15.32)	3.23 (2.62–3.97)	4.04 (2.56–6.38)	0.046
Acrolein	N-Acetyl-S-(3-hydroxypropyl)-L-cysteine (3HPMA)	269.86 (150.96–482.38)	242.36 (201.12–292.05)	302.15 (199.74–457.04)	0.686
	N-Acetyl-S-(2-carboxyethyl)-L-cysteine (CEMA)	95.01 (54.94–164.28)	83.68 (70.18–99.77)	105.81 (71.62–156.31)	0.617
Acrylamide	N-Acetyl-S-(2-carbamoylethyl)-L-cysteine (AAMA)	43.15 (24.16–77.06)	34.59 (28.71–41.67)	51.30 (33.93–77.54)	0.297
	N-Acetyl-S-(2-carbamoyl-2-hydroxyethyl)-L-cysteine (GAMA)	15.38 (9.02–26.21)	10.57 (8.90–12.55)	13.91 (9.50–20.33)	0.330
Acrylonitrile	N-Acetyl-S-(2-cyanoethyl)-L-cysteine (CYMA)	3.75 (1.47–9.56)	2.26 (1.67–3.04)	3.82 (1.96–7.45)	0.368
Benzene	N-Acetyl-S-(phenyl)-L-cysteine (PMA)	0.83 (0.49–1.39)	0.83 (0.70–0.98)	1.07 (0.74–1.54)	0.497
1,3-Butadiene	N-Acetyl-S-(4-hydroxy-2-buten-1-yl)-L-cysteine (MHBMA3)	6.99 (3.67–13.29)	5.75 (4.67–7.06)	7.50 (4.74–11.86)	0.617
	N-Acetyl-S-(3,4-dihydroxybutyl)-L-cysteine (DHBMA)	213.51 (135.60–336.18)	230.58 (199.30–266.76)	248.88 (180.10–343.93)	0.847

Crotonaldehyde	N-Acetyl-S-(3-hydroxypropyl-1-methyl)-L-cysteine (HPMMA)	346.25 (210.09–570.64)	275.75 (234.87–323.72)	328.26 (229.94–468.61)	0.612
Cyanide	2-Aminothiazoline-4-carboxylic acid (ATCA)	95.15 (48.17–187.93)	79.23 (63.68–98.59)	85.68 (52.75–139.15)	0.887
N,N-Dimethylformamide; methyl isocyanate	N-Acetyl-S-(N-methylcarbamoyl)-L-cysteine (AMCC)	144.59 (86.21–242.52)	122.62 (103.85–144.77)	197.13 (136.37–284.96)	0.116
Ethylene oxide; acrylonitrile; vinyl chloride	N-Acetyl-S-(2-hydroxyethyl)-L-cysteine (HEMA)***	0.72 (0.42–1.21)	0.47 (0.40–0.56)	0.55 (0.37–0.80)	0.351
Propylene oxide	N-Acetyl-S-(2-hydroxypropyl)-L-cysteine (2HPMA)	43.80 (23.24–82.52)	36.81 (30.03–45.12)	36.85 (23.46–57.87)	0.881
Styrene	Mandelic acid (MA)	122.76 (74.56–202.20)	107.49 (91.57–126.16)	117.01 (82.00–166.98)	0.866
Styrene, ethylbenzene	Phenylglyoxylic acid (PGA)	118.31 (66.81–209.48)	103.89 (86.47–124.81)	111.23 (74.02–167.12)	0.911
Toluene; benzyl alcohol	N-Acetyl-S-(benzyl)-L-cysteine (BMA)	3.88 (2.12–7.10)	4.36 (3.59–5.30)[c]	8.07 (5.24–12.41)[b]	0.042
Xylene	2-Methylhippuric acid (2MHA)	20.19 (10.70–38.07)	14.14 (11.54–17.34)	16.99 (10.81–26.71)	0.572
	3- + 4-Methylhippuric acids (34MHA)	103.12 (55.98–189.92)	87.60 (72.00–106.58)	99.75 (64.55–154.13)	0.839

Note: [a] indicates statistically significant difference from replaceable cartridge; [b] indicates statistically difference from refillable users; [c] indicates statistically significant difference from "other" device users (all $p < 0.05$). Reported p-values indicate findings from omnibus statistic; superscript letter notation reflects findings adjusted for multiple comparisons (sidak). Geometric means are adjusted for urinary creatinine, age, sex, race, and country of residence.
